# Exploring drug resistance genes in *Acinetobacter baumannii* using metagenomic next-generation sequencing

**DOI:** 10.3389/fmicb.2025.1669208

**Published:** 2025-10-09

**Authors:** Guiqiu Li, Jianxing Lao, Yixiong Jiang, Pei Tang, Hui Huang, Lijuan He, Ke Yuan, Xiulan Lai

**Affiliations:** ^1^Shenzhen Nanshan People’s Hospital and Affiliated Nanshan Hospital of Shenzhen University, Shenzhen, China; ^2^Beijing Genomics Institute (BGI), Shenzhen, China

**Keywords:** *Acinetobacter baumannii*, metagenomic next-generation sequencing, antibiotic resistance genes, *β*-lactam antibiotics, minocycline, aminoglycoside, quinolone

## Abstract

**Introduction:**

With the rising incidence of infectious diseases, the overuse of antibiotics has become a serious problem in clinical practice. In recent years, metagenomic next-generation sequencing (mNGS) has emerged as a promising alternative, offering advantages such as rapid turnaround, broad-spectrum detection, and comprehensive coverage. However, its clinical utility for antimicrobial resistance testing remains to be fully established

**Methods:**

In this study, we evaluated the effectiveness of mNGS in detecting antimicrobial resistance in Acinetobacter baumannii using 53 clinical samples. The performance of mNGS was compared with conventional culture-based methods. In addition, clinical judgment of drug resistance was used as a reference to assess concordance between sequencing results and patient treatment.

**Results:**

Metagenomic sequencing produced an average of 8.4 × 10⁷ reads per sample and identified 61 resistance loci—20 of which appeared in at least five isolates. Among the A. baumannii–positive samples, class-specific accuracy of mNGS exceeded 80% for β-lactams, aminoglycosides, quinolones, and minocycline, underscoring its strong performance in comprehensive resistome profiling. We further investigated resistance-associated genes in A. baumannii that appeared with high frequency, including enzymatic inactivation mechanisms (ADC-type cephalosporinases and OXA-type oxacillinases), efflux systems (AbaQ, AbeM), and RND-type efflux pumps (adeIJK/adeN and adeFGH/adeL).

**Discussion:**

Our findings demonstrate a high concordance between mNGS results, culture-based methods, and clinical evaluations, highlighting the potential of mNGS as a reliable tool for assessing antimicrobial resistance in *A. baumannii.*

## 1 Introduction

*Acinetobacter baumannii* is a non-fermentative, aerobic, Gram-negative bacterium. It is a pathogen that often causes respiratory and bloodstream infections (Garnacho-Montero et al., 2005; Chopra et al., 2013; Antunes et al., 2014). It is well known for its ability to develop multidrug resistance, which creates major treatment challenges. It is a frequent cause of community-acquired infections and also common in intensive care units (Dijkshoorn et al., 2007). Inappropriate and excessive antibiotic use has further accelerated resistance in recent years. Carbapenem-resistant *A. baumannii* (CRAB) has become a major concern among carbapenem-resistant Gram-negative bacteria (Tacconelli et al., 2018; Islam et al., 2023; Afzal-Shah et al., 2001; Mera et al., 2010). Data from the China Antimicrobial Resistance Surveillance Network (2021–2023) show alarmingly high resistance rates of *A. baumannii* to both imipenem and meropenem (CHINET, n.d.).

Currently, the detection of antibiotic resistance in *Acinetobacter baumannii* within hospital settings relies mainly on culture-based methods. However, these methods are limited by long turnaround times, complex procedures, and low positive rates (Miao et al., 2018). While numerous reports have explored drug susceptibility prediction models based on whole genome sequencing (WGS), the technical intricacies of isolating and purifying bacterial samples restrict widespread clinical application (Cohen et al., 2019). mNGS offers distinct advantages, including high positivity rates, minimal interference from antibacterial agents, and broad pathogen coverage (Gu et al., 2019). It has extensive utility in clinical diagnosis and treatment (Lu et al., 2022; Liu et al., 2022). By leveraging database-matched resistance genes, mNGS might characterize the drug resistance profiles of bacteria, providing valuable insights to guide the management of infectious diseases (Hu et al., 2023; Serpa et al., 2022).

In this study, we evaluated the efficacy of mNGS technology for detecting drug resistance in *Acinetobacter baumannii* and compared its performance with conventional culture-based methods. We further investigated the correlation between antibiotic resistance genes and phenotypic resistance to assess the concordance between mNGS and antimicrobial susceptibility testing (AST). In addition, we examined the accuracy of mNGS in detecting resistance across multiple antibiotic classes, including *β*-lactams, aminoglycosides, fluoroquinolones, and minocycline.

## 2 Materials and methods

### 2.1 Research objects

From July 2021 to October 2023, samples from adult patients were continuously collected in the Laboratory Department of Union Shenzhen Hospital, Huazhong University of Science and Technology. A total of 754 samples from 628 cases underwent paired testing using both mNGS and culture-based methods, and 53 samples from 42 patients were confirmed positive for *Acinetobacter baumannii* by either method. The inclusion criteria were as follows: (1) age ≥18 years; (2)clinical manifestations: Presence of infection-related symptoms such as fever, cough, chest tightness, or dyspnea, consistent with *A. baumannii* infections such as respiratory tract and bloodstream infections; (3)imaging/laboratory evidence: For pulmonary infections, chest CT demonstrating inflammatory infiltrates; for bloodstream infections, laboratory tests indicating elevated white blood cell counts or procalcitonin (PCT) levels; and (4)samples tested by both mNGS and conventional microbiological methods. The exclusion criteria included duplicate short-term samples from the same patient in which the antimicrobial resistance status was clinically judged to be unchanged, cases in which the interval between mNGS and culture-based methods exceeded 1 week, and specimens lacking complete clinical information. The study was conducted in accordance with the Declaration of Helsinki, and the protocol was approved by the Ethics Committee of Union Shenzhen Hospital, Huazhong University of Science and Technology (approval number: LW-2024-005).

### 2.2 Sample preparation

For plasma samples, 3 mL of peripheral blood was collected, left at room temperature for 3–5 min, and centrifuged within 8 h at 4,000 rpm for 10 min at 4 °C. The plasma samples were then transferred to sterile tubes. For sputum and BALF samples, 1.5–3 mL specimens were collected according to standard procedures. A 0.45 mL aliquot was treated with saponin (final concentration 0.025%), vortexed for 15 s, and incubated at 25 °C for 5 min. After adding 75 μL of host depletion reagent, the mixture was vortexed again and incubated at 37 °C for 10 min, followed by centrifugation at 18,000 g for 5 min. The supernatant was partially removed, PBS wash steps were performed, and the pellet was resuspended in 370 μL of TE buffer. Lysozyme (7.2 μL) was added for cell wall lysis, followed by bead beating with 250 μL of 0.5 mm glass beads at 2800–3200 rpm for 30 min. DNA was extracted from 300 μL of the resulting solution using the TIANamp Micro DNA Kit (DP316, TIANGEN BIOTECH, Beijing, China) according to the manufacturer’s instructions. The extracted DNA specimens were used for the construction of DNA libraries (Long et al., 2016).

### 2.3 Construction of libraries and sequencing

Genomic DNA was isolated from the specimens using the TIANMicrobe Magnetic Bead Pathogenic Microbial DNA Extraction Kit (catalog No. NG550-01) in accordance with the protocols provided by the manufacturer. The resulting DNA underwent a series of manipulations, including enzymatic digestion, end repair, adapter ligation, and PCR amplification, to generate sequencing libraries. The Agilent 2,100 Bioanalyzer was utilized to verify that the fragment sizes were approximately 300 bp, while the Qubit dsDNA HS Assay Kit (Thermo Fisher Scientific) was used to determine library concentrations. Each library concentration was adjusted based on the necessary sequencing depth. Subsequently, the libraries were combined and subjected to a circularization reaction to form circular DNA molecules. DNA nanoballs (DNBs) were synthesized via rolling circle amplification (RCA), and these DNBs were loaded onto a microfluidic chip for sequencing using the BGIseq platform. After sequencing, the samples were de-duplicated using barcodes, and data quality was ensured by checking sequencing depth and carrying out quality control procedures (Jeon et al., 2014).

### 2.4 Culture-based method and antimicrobial susceptibility testing

Fresh samples from the participants were collected and retained at Shenzhen Hospital of Huazhong University of Science and Technology under the supervision of professional staff. The samples were separately streaked onto blood agar plates, MacConkey plates, and chocolate plates with sterile cotton swabs and placed in a CO2 bacterial incubator (SANYO, Japan) for 24 h. Suspected pathogenic colonies were selected from each plate afterward. According to the manufacturer’s instructions, isolates were identified using an automated rapid biological mass spectrometry system (IVD MALDI Biotyper).

The identified isolates were further inoculated onto agar plates for purification and placed in a CO2 incubator (SANYO, Japan) for 24 h. Antimicrobial susceptibility testing of the purified isolates was performed using an automated microbial identification and drug sensitivity analyzer (BD, USA) with either a Gram-positive bacterial drug sensitivity plate (BD PhoenixTM PMIC-92) or a Gram-negative bacterial drug sensitivity plate (BD PhoenixTM NMIC-413). The outcome was interpreted according to the manufacturer’s instructions and the American Council for Clinical Laboratory Standards (CLSI, M100).

### 2.5 Bioinformatic analysis

First, raw sequencing data generated by the BGISeq platform were preprocessed using the fastp software to remove low-quality reads and trim adapter sequences (Chen et al., 2018). The resulting high-quality sequences after filtration were referred to as “Clean data.” Next, the Clean data were mapped to the human genome (GRCh38) using the Burrows-Wheeler alignment (BWA) software (Li and Durbin, 2009), followed by stripping to obtain annotated human genomic data. The remaining sequencing data were aligned against the microbial genomes in the Pathogenic Metagenomics Database (PMDB), which includes genomes of viruses, bacteria, fungi, and parasites associated with human diseases. Specifically, the PMDB contains sequence data from 10,830 bacterial, 5,050 viral, 1,179 fungal, 282 parasitic, and 159 mycoplasma/chlamydia strains, with all reference genomes downloaded from the National Center for Biotechnology Information (NCBI) (Wang et al., 2022). The criteria for obtaining metagenomic next-generation sequencing (mNGS) data were followed according to the protocol described by Ren et al. (2021).

For the samples positive by mNGS, two comparative analyses were performed using the Clean data. First, the Hisat2 software was utilized to exclude human (GRCh38) gene sequences (Kim et al., 2019). Subsequently, the remaining sequences were mapped to the CARD database, with resistance genes identified using the Resistance Gene Identifier (RGI) software (Alcock et al., 2023). From these identification results, we screened for resistance genes localized to *Acinetobacter baumannii*, retaining only those with a mapping quality ≥ 0 and allele coverage ≥ 5% for subsequent analysis.

### 2.6 Statistical analysis

The demographic characteristics of all enrolled patients were summarized and presented as median (95% confidence interval [CI]) and count (inclusion proportion). The chi-squared test was used to compare the detection rates of mNGS and the culture-based method, with a *p*-value of < 0.05 considered statistically significant. The chi-squared test was performed using GraphPad Prism 8.0. All graphs were generated using the R (version 4.2.3) package ggplot2, as well as GraphPad Prism 8.0.

## 3 Results

### 3.1 Study design

We analyzed 754 samples from 628 cases, all of which underwent paired testing by mNGS and conventional culture. Among samples in which *A. baumannii* was determined to be the primary pathogen, 701 samples (92.97%) were negative for *A. baumannii* by both methods, while 19 samples (2.52%) were positive by both. However, some discrepancies were observed: 30 samples (3.98%) were positive only by mNGS, suggesting higher sensitivity of sequencing, whereas four samples (0.53%) were positive only by culture. Taken together, a total of 53 samples were identified as *A. baumannii* positive by either method, and these were included in the subsequent analyses.

**Figure1:**
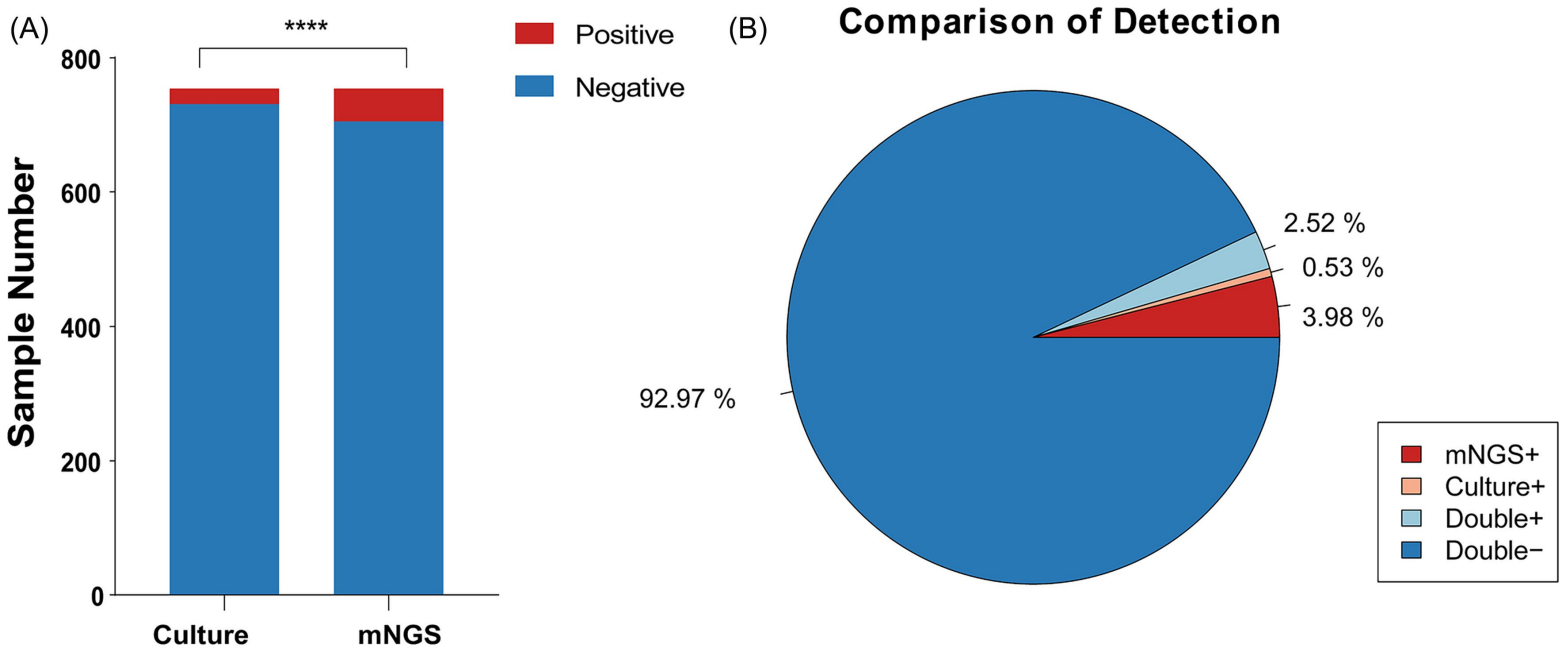
Positivity rate comparison and concordance analysis of mNGS and culture-based methods in *Acinetobacter baumannii* Infection. **(A)** The number of positive samples (y-axis) for pairwise mNGS and culture-based method testing. **(B)** The pie chart illustrating the positivity rate distribution between mNGS and culture-based methods.

The demographic and clinical characteristics of the patients with suspected *Acinetobacter baumannii* infection are provided in Table 1. As shown in the table, 26 cases (61.90%) were older than 65 years, and the median age was 72 years. In terms of clinical manifestations, fever was observed in 22 patients (52.38%), cough in 11 patients (26.19%), difficulty breathing in six patients (14.29%), chest tightness and shortness of breath in four (9.52%) patients, and chest pain in one patient (2.38%). Within this patient group, 16 (38.10%) individuals were hypertensive, eight (19.05%) had diabetes, seven (16.67%) were diagnosed with cancer, five (11.90%) had coronary heart disease, and four (9.52%) had hyperglycemia and experienced chronic obstructive pulmonary disease. The clinical outcomes revealed that 22 patients were discharged after recovery, 15 patients unfortunately passed away, and the status of five patients remains undetermined (see Figure 1).

**Table 1:** Demographic and clinical characteristics of the patients.

Demographic and clinical characteristics	Patient [*n* (%)]
Patient	42
Age: year	
18–65	16 [38.10%]
>65	26 [61.90%]
Median [region of 95% CI]	72 [59–76]
Temperature	
Median [region of 95% CI]	36.6 [36.5–37.1]
Pulse	
Median [region of 95% CI]	93 [89–102]
Breathe	
Median [region of 95% CI]	57.3 [26.86–83.96]
CRP	
Median [region of 95% CI]	80.52 [34.56–158.4]
Clinical features	
Fever	22 [52.38%]
Cough	11 [26.19%]
Chest tightness and shortness of breath	6 [14.29%]
Difficulty breathing	4 [9.52%]
Chest pain	1 [2.38%]
Underlying medical conditions	
Hypertension	16 [38.10%]
Diabetes	8 [19.05%]
Tumor	7 [16.67%]
Coronary heart disease	5 [11.90%]
High uric acid	4 [9.52%]
Chronic obstructive pulmonary disease	4 [9.52%]
Duration of treatment	
Median [region of 95% CI]	24 [19–45]
Outcome	
Recover	22 [52.38%]
Death	15 [35.71%]
Other	5 [11.90%]

### 3.2 Comparison of the detection of microorganisms by mNGS and culture-based methods

**Figure 2:**
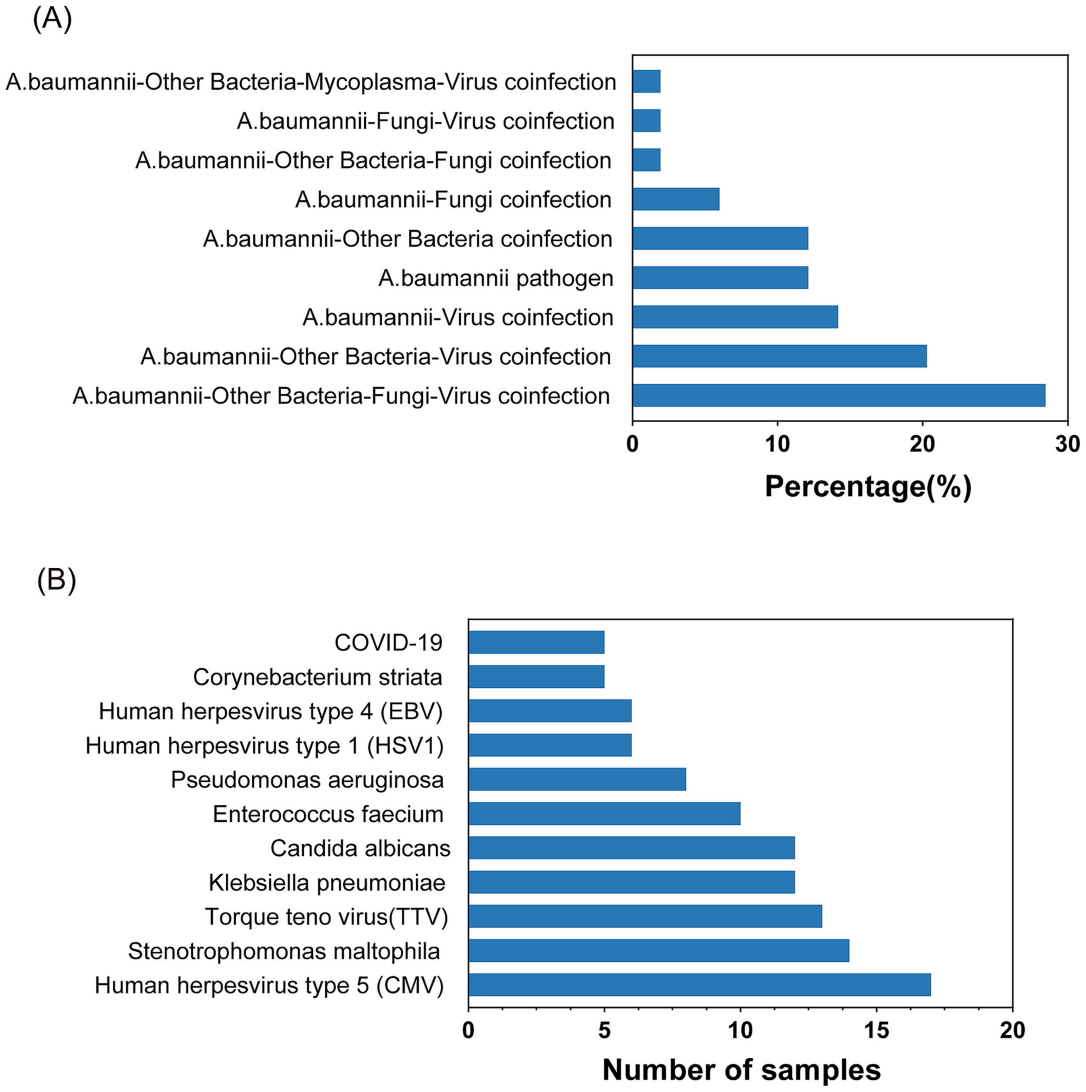
Statistics of *Acinetobacter baumannii* infection. **(A)** Common combinations of mixed infections. **(B)** Top pathogens among various microbes in co-infections.

The mNGS detection of *Acinetobacter baumannii* was conducted on 53 samples, revealing a significant number of co-infections. As depicted in Figure 2A, 12.24% of the samples were infected with *Acinetobacter baumannii* only. The remaining 87.76% of the samples exhibited co-infections involving *Acinetobacter baumannii* along with other bacteria, fungi, and viruses. Among the 47 co-infection samples, the highest rate was attributed to samples co-infected with *Acinetobacter baumannii*, other bacteria, fungi, and viruses, constituting 28.57%. This was followed by samples co-infected with *Acinetobacter baumannii,* other bacteria, and viruses at 20.41%. Figure 2B illustrates the top 11 pathogens identified among the co-infected samples, with *Stenotrophomonas maltophilia* and *Klebsiella pneumoniae* being the most prevalent bacteria. The viruses most frequently co-infecting with *Acinetobacter baumannii* were human herpesvirus type 5 (CMV) and Torque teno virus (TTV), and the fungus with the highest co-infection rate was *Candida albicans*.

### 3.3 Resistance gene detection statistics and analysis of mNGS

**Figure 3:**
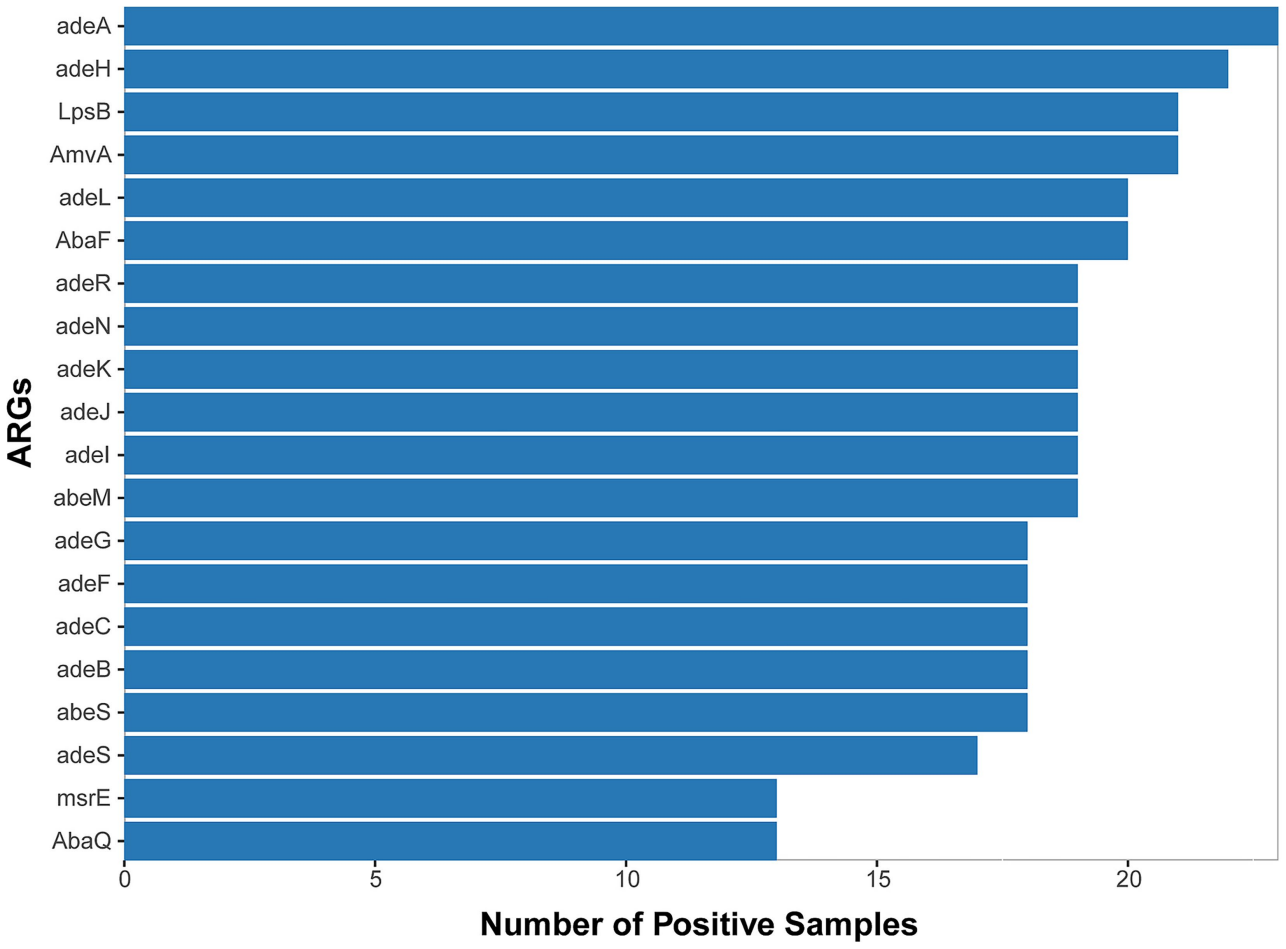
Top 20 ARGs detected from *53 A. baumannii*-positive samples (mNGS/culture-based methods) across 30 cases with positive detection.

We ultimately conducted a comparative analysis of drug resistance in 53 samples from 42 different patients that tested positive by both mNGS and culture-based methods. A mean of 8.4 ×10^7^ sequence reads was generated by mNGS. In total, approximately 61 distinct resistance genes of *Acinetobacter baumannii* were identified from 30 samples through annotation against the CARD database (Supplementary Table 1 and Supplementary Figure 1), and the top 20 genes are shown in Figure 3. We observed that each of these main antimicrobial resistance genes was present in over five samples. Among these, RND-type efflux pump systems were the most prevalent category. Specifically, the adeIJK operon (and its regulator adeN), which confers resistance to *β*-lactams, fluoroquinolones, and tetracyclines, was detected in the majority of the samples. Similarly, the adeFGH genes under the control of the adeL regulator—both known to mediate fluoroquinolone and tetracycline resistance—were also frequently identified (Damier-Piolle et al., 2008; Oh et al., 2017; Kaviani et al., 2020; Coyne et al., 2010). The adeAB efflux pump and its two-component regulatory system adeRS, classically associated with tetracycline resistance, were also commonly observed. Finally, members of the MATE transporter family, notably AbaQ and AbeM—which facilitate fluoroquinolone extrusion—were detected across the cohort (Xu et al., 2019; Wu et al., 2023).

Beyond efflux mechanisms, enzyme-mediated resistance determinants also emerged as a prominent contributor to antibiotic resistance. Specifically, two key classes of *β*-lactamases were identified in a substantial proportion of the isolates: the *Acinetobacter*-derived cephalosporinase (ADC) family, which mediates the hydrolysis of cephalosporins, and OXA-type oxacillinases, responsible for the hydrolysis of carbapenems. Notably, we detected several genes belonging to the OXA-51 class—currently recognized as the largest subgroup of OXA-type β-lactamases—including OXA-79, OXA-121, OXA-128, OXA-131, and OXA-148. These OXA-51 class enzymes are intrinsic to *Acinetobacter baumannii*, with their encoding genes naturally located on the bacterial chromosome. Importantly, under significant selective pressure imposed by antibiotic use, these chromosomally encoded enzymes play a meaningful role in driving drug resistance. This observation suggests that β-lactamases of this class may contribute to drug resistance in *Acinetobacter baumannii* (*A. baumannii*) (Evans and Amyes, 2014). Collectively, these findings reveal a multifaceted resistome in *Acinetobacter baumannii*, combining broad-spectrum efflux systems with specialized enzymatic inactivation of key antibiotic classes.

### 3.4 Comparison between mNGS and phenotypic antimicrobial susceptibility testing

**Figure 4:**
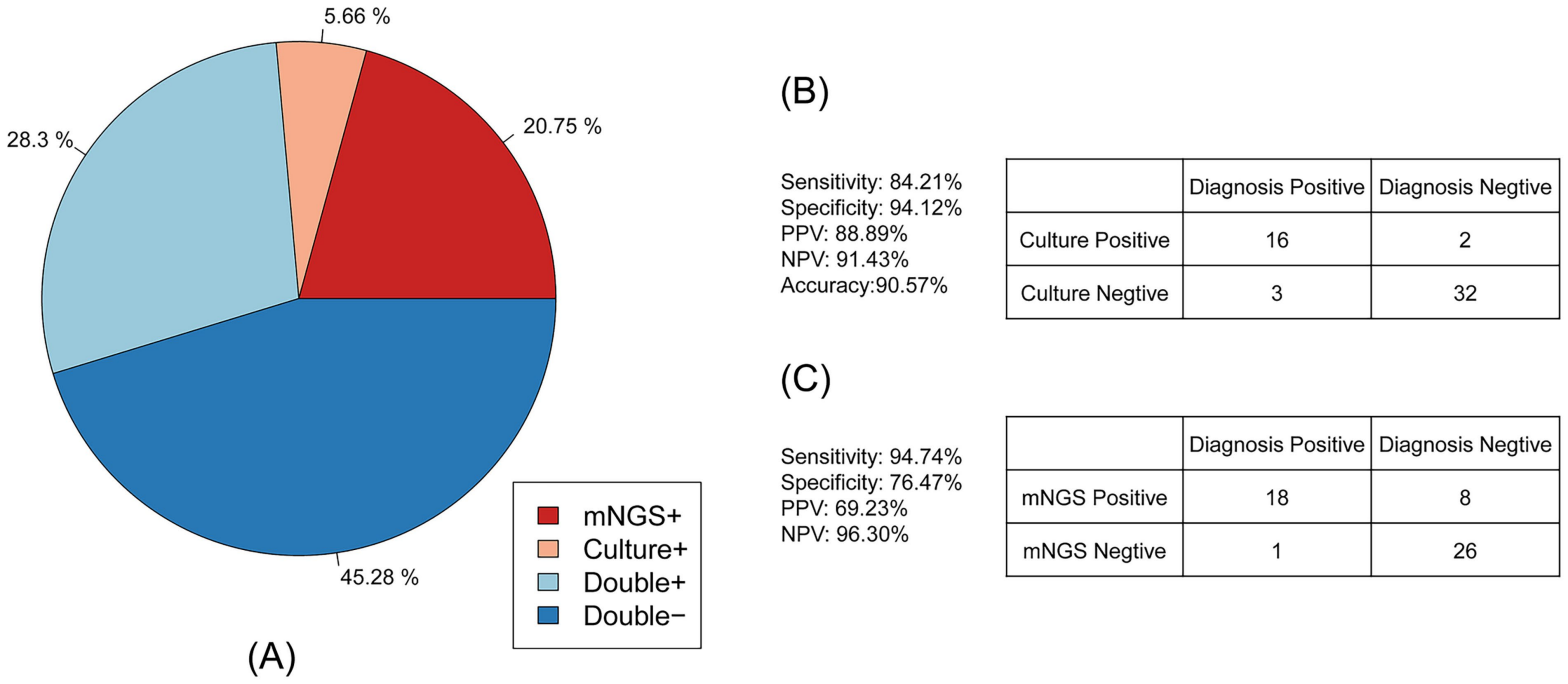
Consistency of the *Acinetobacter baumannii* drug resistance results between mNGS and culture-based methods. **(A)** The pie chart showing the concordance of the antimicrobial resistance results between mNGS and culture-based methods. **(B)** Contingency tables formatted in a 2 × 2 manner showing the drug resistance performance of culture-based method testing for clinical diagnosis. **(C)** Contingency tables formatted in a 2 × 2 manner showing the drug resistance performance of mNGS for clinical diagnosis.

Antimicrobial susceptibility testing for the phenotypic characterization of *Acinetobacter baumannii* was also performed on 53 samples. The consistency between antibiotic resistance genes and phenotypes is shown in Figure 4A. A total of 28.30% of samples were drug-resistant according to both mNGS and antimicrobial susceptibility testing, 45.28% were negative by both methods, 20.75% were detected only by mNGS, and 5.66% were detected only by antimicrobial susceptibility testing. These results indicate that mNGS has high accuracy in detecting drug resistance.

As shown in Figure 4B, compared to the clinical diagnosis, traditional antimicrobial (culture result) susceptibility testing showed high accuracy of up to 90.57%. The sensitivity of antimicrobial susceptibility testing was 84.21%, and the specificity was 94.12%. The positive predictive value (PPV) of antimicrobial susceptibility testing was 88.89%, and the negative predictive value (NPV) was 91.43%. For the mNGS drug resistance result described in Figure 4C, the sensitivity was up to 94.74% and the NPV was 96.30%, while the specificity and PPV were 76.47 and 69.23%, respectively. The accuracy of mNGS was only 83.02%.

In addition, we calculated the sensitivity and specificity of mNGS for different types of antibiotics, which are shown in Table 2. Of the 53 samples, 17 exhibited resistance to *β*-lactam antibiotics, 13 to aminoglycosides, 15 to fluoroquinolones, 16 to minocycline, and four to tigecycline in the clinical diagnosis. The sensitivity for β-lactam antibiotic resistance was 89.47%, which was higher than that for aminoglycosides (72.22%), fluoroquinolones (83.33%), minocycline (88.89%), and tigecycline (57.14%). The specificity for aminoglycosides was highest, reaching up to 88.57%, while the specificity for β-lactam antibiotics was 85.29%, fluoroquinolones was 80%, minocycline was 77.14%, and tigecycline was 71.74%. The PPV, NPV, and accuracy for *β*-lactam antibiotics (77.27% for PPV, 93.55% for NPV, 86.79% for accuracy) were higher than those for aminoglycosides (76.47% for PPV, 86.11% for NPV, 83.02% for accuracy), fluoroquinolones (68.18% for PPV, 90.32% for NPV, 81.13% for accuracy), minocycline (66.67% for PPV, 93.10% for NPV,81.13% for accuracy), and tigecycline (23.53% for PPV, 91.67% for NPV, 69.81% for accuracy).

**Table 2:** *Acinetobacter baumannii* susceptibility or resistance to antibiotics.

Type	Antibiotics	No. of true resistance	No. of false resistance	No. of true susceptibility	No. of false susceptibility	Sensitivity (%)	Specificity (%)	PPV (%)	NPV (%)	Accuracy (%)
Culture-based method	β-lactam	17	0	34	2	89.47	100.00	100.00	94.44	96.23
	Aminoglycoside	12	2	33	6	66.67	94.29	85.71	84.62	84.91
	Quinolone	15	2	33	3	83.33	94.29	88.24	91.67	90.57
	Minocycline	11	2	33	7	61.11	94.29	84.62	82.50	83.02
	Tigecycline	0	1	45	7	0.00	97.83	0.00	86.54	84.91
mNGS	β-lactam	17	5	29	2	89.47	85.29	77.27	93.55	86.79
	Aminoglycoside	13	4	31	5	72.22	88.57	76.47	86.11	83.02
	Quinolone	15	7	28	3	83.33	80.00	68.18	90.32	81.13
	Minocycline	16	8	27	2	88.89	77.14	66.67	93.10	81.13
	Tigecycline	4	13	33	3	57.14	71.74	23.53	91.67	69.81

True resistance: Phenotypic antimicrobial susceptibility testing shows resistance and genotypic resistance is predicted.

False resistance: Phenotypic antimicrobial susceptibility testing shows susceptibility and genotypic resistance is predicted.

True susceptibility: Phenotypic antimicrobial susceptibility testing shows susceptibility and genotypic susceptibility is predicted.

False susceptibility: Phenotypic antimicrobial susceptibility testing shows resistance and genotypic susceptibility is predicted.

Table 2 also shows the sensitivity and specificity of traditional antimicrobial susceptibility testing for different types of antibiotics compared to clinical diagnosis. Of the 53 samples, 17 exhibited resistance to β-lactam antibiotics, 12 to aminoglycosides, 15 to fluoroquinolones, and 11 to minocycline, while no samples showed resistance to the tigecycline drug. The specificity for all antibiotics was over 90%, while the NPV and accuracy for all antibiotics were over 80%. In addition, the PPV for all antibiotics, except tigecycline, was also over 80%. While the specificity for aminoglycosides was only 66.67% and the specificity for minocycline was only 61.11%, the specificity for tigecycline was zero. Among these antibiotics, β-lactam antibiotics showed better consistency with clinical diagnosis.

## 4 Discussion

*Acinetobacter baumannii* is a significant opportunistic pathogen commonly found in community and hospital settings and is recognized as a critical multidrug-resistant microorganism globally, presenting a substantial challenge to treatment protocols (Antunes et al., 2014). Traditional microbial detection methods often fall short in terms of sensitivity, specificity, and rapidity (Li et al., 2020). In contrast, mNGS offers the capability to analyze the full spectrum of microorganisms present in patient samples, making it an invaluable tool for pathogen detection in critically ill patients. In our study, we assessed the effectiveness of mNGS and conventional culture-based methods in detecting *Acinetobacter baumannii* in 42 patients. Furthermore, in samples where *Acinetobacter baumannii* was identified by both mNGS and culture-based methods, we analyzed the antibiotic resistance genes associated with common drug-resistant strains of *Acinetobacter baumannii*. In addition, we examined the concordance between the drug resistance profiles and clinical outcomes of *Acinetobacter baumannii* as determined by mNGS and culture-based methods.

In this study, the mNGS analysis of 53 clinical specimens revealed that mono-infection with *Acinetobacter baumannii* was relatively uncommon, accounting for only 12.24% of cases, while the vast majority (87.76%) harbored one or more additional pathogens. Notably, mixed bacterial–fungal–viral infections represented the single largest category of co-infections (28.57%), underscoring the complex microbial milieu encountered in critically ill patients. The high prevalence of *Stenotrophomonas maltophilia* and *Klebsiella pneumoniae* alongside *Acinetobacter baumannii* likely reflects shared ecological niches in hospital environments and overlapping risk factors, such as invasive devices and broad-spectrum antibiotic exposure. Similarly, the detection of opportunistic viruses (e.g., CMV, TTV) and fungi (e.g., *Candida albicans*) highlights the vulnerability of these patients to reactivation or colonization events that may complicate both diagnosis and treatment (Wang et al., 2020; Wang et al., 2019; Han et al., 2020; Mouton et al., 2020). Importantly, mNGS offers the capacity to detect multiple pathogens simultaneously and to uncover fastidious or unexpected co-infecting organisms that conventional culture-based methods often miss. This broader diagnostic insight can inform more tailored antimicrobial regimens and prompt consideration of antiviral or antifungal therapies, thereby potentially improving patient outcomes.

The high concordance observed between mNGS-based resistance gene detection and phenotypic antimicrobial susceptibility testing (95.58% overall agreement) underscores the potential of mNGS as a reliable tool for comprehensive resistome profiling in *A. baumannii* infections (Lu et al., 2022; Serpa et al., 2022). Notably, mNGS demonstrated superior sensitivity (94.74%) for identifying resistance determinants compared to the traditional methods (84.21%), although its specificity (76.47%) and positive predictive value (69.23%) were lower, indicating that mNGS may detect low-level or non-expressed genes that do not translate into phenotypic resistance (Holt et al., 2016; Jo and Ko, 2021). Among RND-type efflux pumps, adeIJK, a fundamental system ubiquitous across *Acinetobacter* spp.,and its regulator adeN were nearly universal, mediating resistance to *β*-lactams, fluoroquinolones, and tetracyclines (Serpa et al., 2022; de Abreu et al., 2021). The overexpression of the adeABC operon, often driven by mutations in the sensor kinase AdeS, contributed substantially to multidrug resistance, including decreased susceptibility to tigecycline (Yang et al., 2019; Novović et al., 2024). Enzymatic inactivation mechanisms were similarly prominent: ADC variants conferred high-level resistance to extended-spectrum cephalosporins (Curtis et al., 2020; Rodríguez-Martínez et al., 2010), while OXA-type oxacillinases (notably the bla_OXA-23, −24, −51, and −58 alleles) underpinned carbapenem resistance in the majority of isolates (Evans and Amyes, 2014; Li et al., 2023). Finally, MATE family transporters such as AbaQ and AbeM were detected in several samples, indicating a role in fluoroquinolone efflux and further broadening the resistome landscape (Abdi et al., 2020; Vanessa Kornelsen, 2021). Together, these findings highlight the multifactorial nature of antimicrobial resistance in *A. baumannii* and support the integration of mNGS into routine diagnostics to capture both canonical and emerging resistance determinants.

Efflux systems also contribute to resistance. MATE family transporters such as AbeM—an H^+^-coupled multidrug efflux pump encoded by the abeM gene—extrude quinolones, aminoglycosides, and toxic compounds, while AbaQ, a recently identified efflux pump, has been functionally validated to confer quinolone resistance through active extrusion (Abdi et al., 2020; Vanessa Kornelsen, 2021; Marchand et al., 2004; Su et al., 2005; Pérez-Varela et al., 2018). The detection of these transporters across multiple isolates highlights the contribution of diverse efflux systems in broadening resistance profiles.

Enzyme-mediated inactivation has also been a prominent mechanism. ADC variants conferred resistance to extended-spectrum cephalosporins (Novović et al., 2024; Curtis et al., 2020), although they were present in a relatively small proportion of isolates. By contrast, OXA-type oxacillinases—particularly bla < sub > OXA-23</sub>, −24, −51, and −58—were widespread and represented the major contributors to carbapenem resistance (Rodríguez-Martínez et al., 2010; Li et al., 2023). Several alleles of the OXA-51 class, including OXA-79, OXA-121, OXA-128, OXA-131, and OXA-148, were also identified. These chromosomally encoded enzymes are intrinsic to *A. baumannii* and, under the selective pressure of antibiotic use, may play an increasingly important role in driving carbapenem resistance (Rodríguez-Martínez et al., 2010).

Among the resistance mechanisms, RND-type efflux pump systems were the most prevalent category. The AdeIJK system—a broad-spectrum and highly conserved three-component pump encoded by adeI, adeJ, and adeK—together with its regulator adeN, was nearly ubiquitous across *Acinetobacter* spp., mediating resistance to *β*-lactams, tetracyclines, erythromycin, and fluoroquinolones (Serpa et al., 2022; Damier-Piolle et al., 2008; Jo and Ko, 2021). Functional studies using plasmid/strain construction, transcriptional analysis, and mutational screening further identified AdeRS as an essential regulator of adeABC, with specific mutations activating efflux expression (Marchand et al., 2004). In addition, adeFGH—another RND efflux system, typically silent under normal conditions—was shown to mediate resistance to fluoroquinolones and tetracycline/tigecycline when activated by mutations in its upstream LysR-type regulator AdeL, even in strains already harboring adeABC and adeIJK (Coyne et al., 2010). Together, these systems demonstrate how multiple RND efflux pumps interact to expand the intrinsic and acquired resistome of *A. baumannii.*

This study has several limitations. First, the sample size was relatively small, and the study was retrospective and single-center, which may limit generalizability. Second, standardized thresholds for interpreting mNGS-based resistome data were lacking. The criteria used here may not be optimal. Third, the expression of certain resistance determinants, particularly efflux pump–associated genes, can be affected by antibiotic exposure and drug concentrations. Future prospective multicenter studies with larger cohorts are warranted. Such studies will help validate these findings, refine interpretation criteria, and clarify the impact of drug-induced gene expression on the clinical utility of mNGS for antimicrobial resistance profiling.

## 5 Conclusion

In conclusion, our findings demonstrate that mNGS achieves high concordance with phenotypic antimicrobial susceptibility testing and clinical evaluations in profiling drug resistance of *Acinetobacter baumannii*. With predictive values for β-lactams, aminoglycosides, fluoroquinolones, and minocycline, mNGS shows considerable promise as a robust adjunct to conventional methods for guiding antimicrobial therapy. Importantly, future integration of artificial intelligence and machine learning has the potential to enhance interpretive frameworks, enable more comprehensive resistome characterization, and ultimately support the advancement of precision antimicrobial stewardship.

## Data Availability

The original contributions presented in the study are publicly available. This data can be found here: https://db.cngb.org/data_resources/project/CNP0008086/.
