# Supplementary figures and images for: Exploring drug resistance genes in Acinetobacter baumannii using metagenomic next-generation sequencing
Source: Front Microbiol. 2025 Oct 9;16:1669208. doi: 10.3389/fmicb.2025.1669208 (PMC12546095; doi:10.3389/fmicb.2025.1669208)

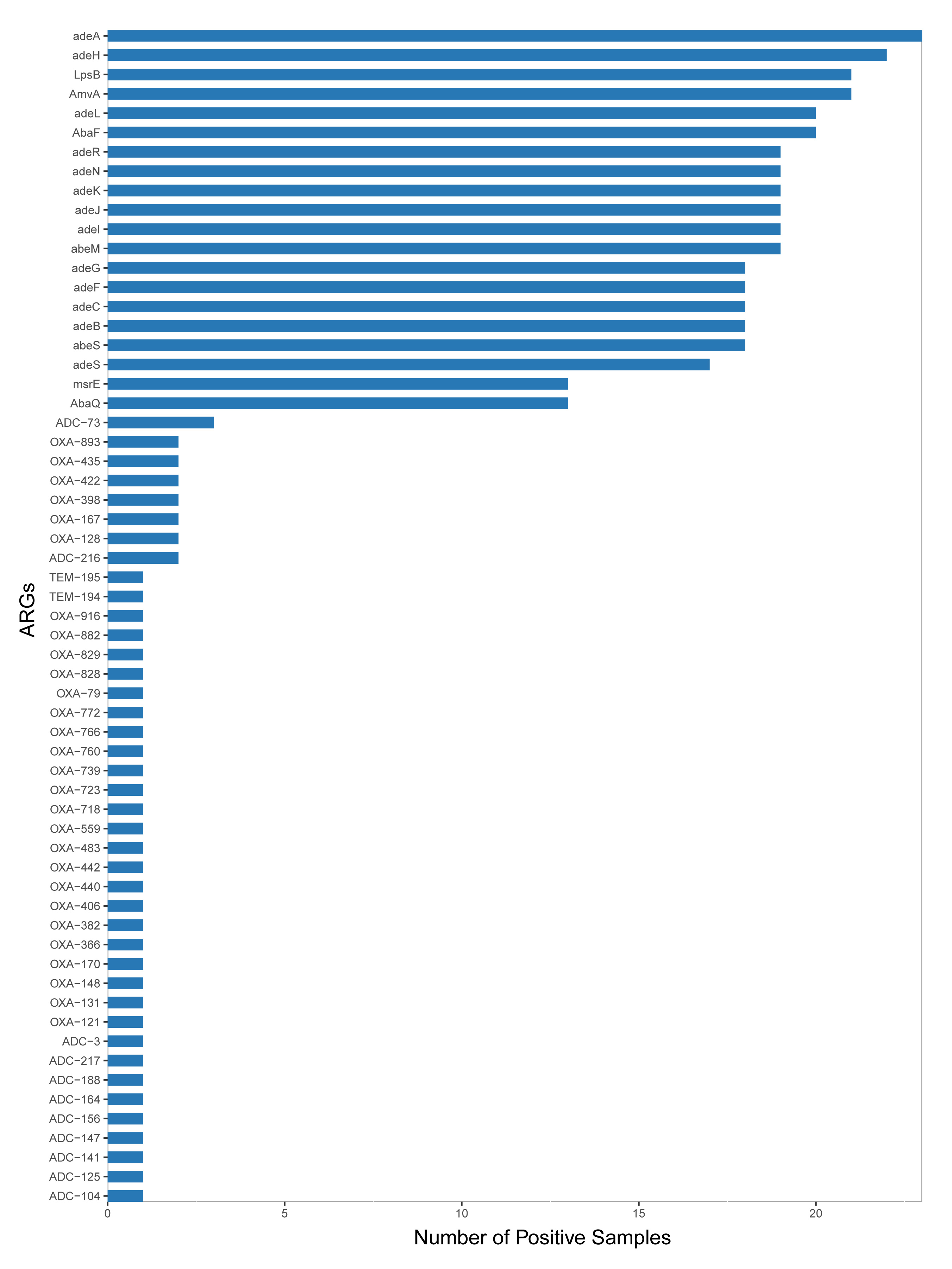

Supplement: Supplementary file 1 [file Image_1.JPEG]
